# Supplementary material for: Flow-driven lumen remodeling and valve opening in the vas deferens
Source: Proc Natl Acad Sci U S A. 2026 Sep 8;123(37):e2538135123. doi: 10.1073/pnas.2538135123 (PMC13578819; doi:10.1073/pnas.2538135123)
Supplement: Supplementary file 1 [file pnas.2538135123.sapp.pdf]

PNAS

Supporting Information for
Flow-driven lumen remodeling and valve opening in the vas deferens

Nicole Ng Shu Ying[1], Qin Ying Lim[1], Gen Yamada[2], *Tsuyoshi Hirashima[1,3,4]

1. Mechanobiology Institute, National University of Singapore, 5A Engineering Drive 1, Singapore, 117411, Singapore
2. Department of Developmental Genetics, Wakayama Medical University, Kimiidera 811-1,Wakayama City, Wakayama 641-8509, Japan
3. Department of Physiology, Yong Loo Lin School of Medicine, National University of Singapore, 2 Medical Drive MD9, Singapore, 117593, Singapore
4. The Hakubi Center, Graduate School of Biostudies, Kyoto University, Yoshida-honmachi, Kyoto, 606-8501, Japan

Corresponding author: Tsuyoshi Hirashima
Email: thira@nus.edu.sg

This PDF file includes:

Figures S1 to S3
Legends for Movies S1 to S14

Other supporting materials for this manuscript include the following:

Movies S1 to S14

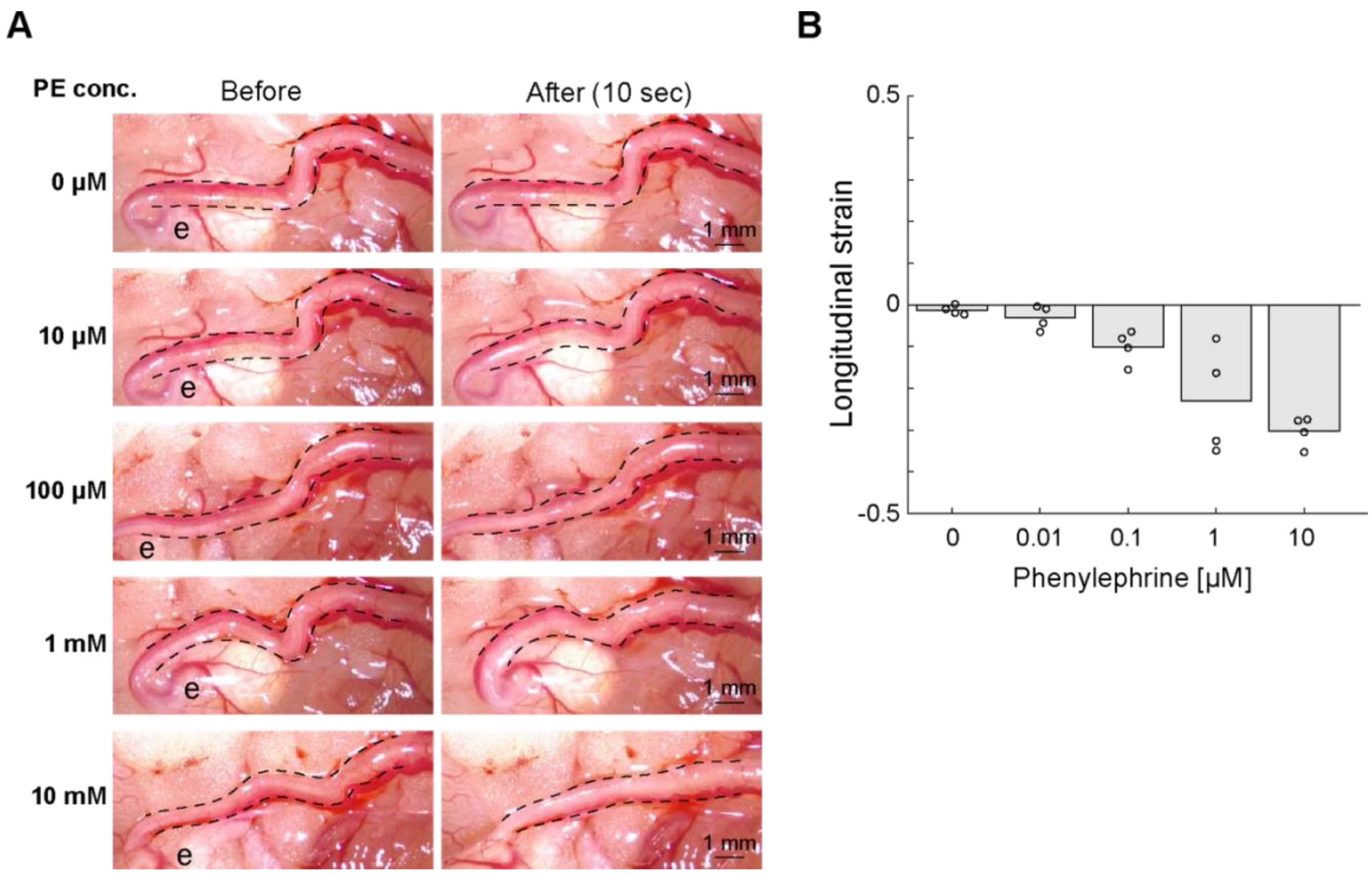

Fig. S1. Dose–response analysis of vas deferens morphological changes induced by phenylephrine.

(A) Representative morphological changes of the vas deferens before (left) and 10 s after treatment (right) under in vivo conditions in anesthetized mice. Phenylephrine (PE) was applied at concentrations of 0 μM (PBS control), 10 μM, 100 μM, 1 mM, and 10 mM. Black dotted lines outline the outer surface of the vas deferens, and "e" indicates the cauda epididymis. Scale bars, 1 mm.

(B) Quantification of longitudinal strain, defined as the relative change in length before and after PE treatment under ex vivo conditions. Statistical significance was assessed using the Jonckheere–Terpstra trend test (one-sided), $p = 7.9 \times 10^{-6}$. N = 4 per condition.

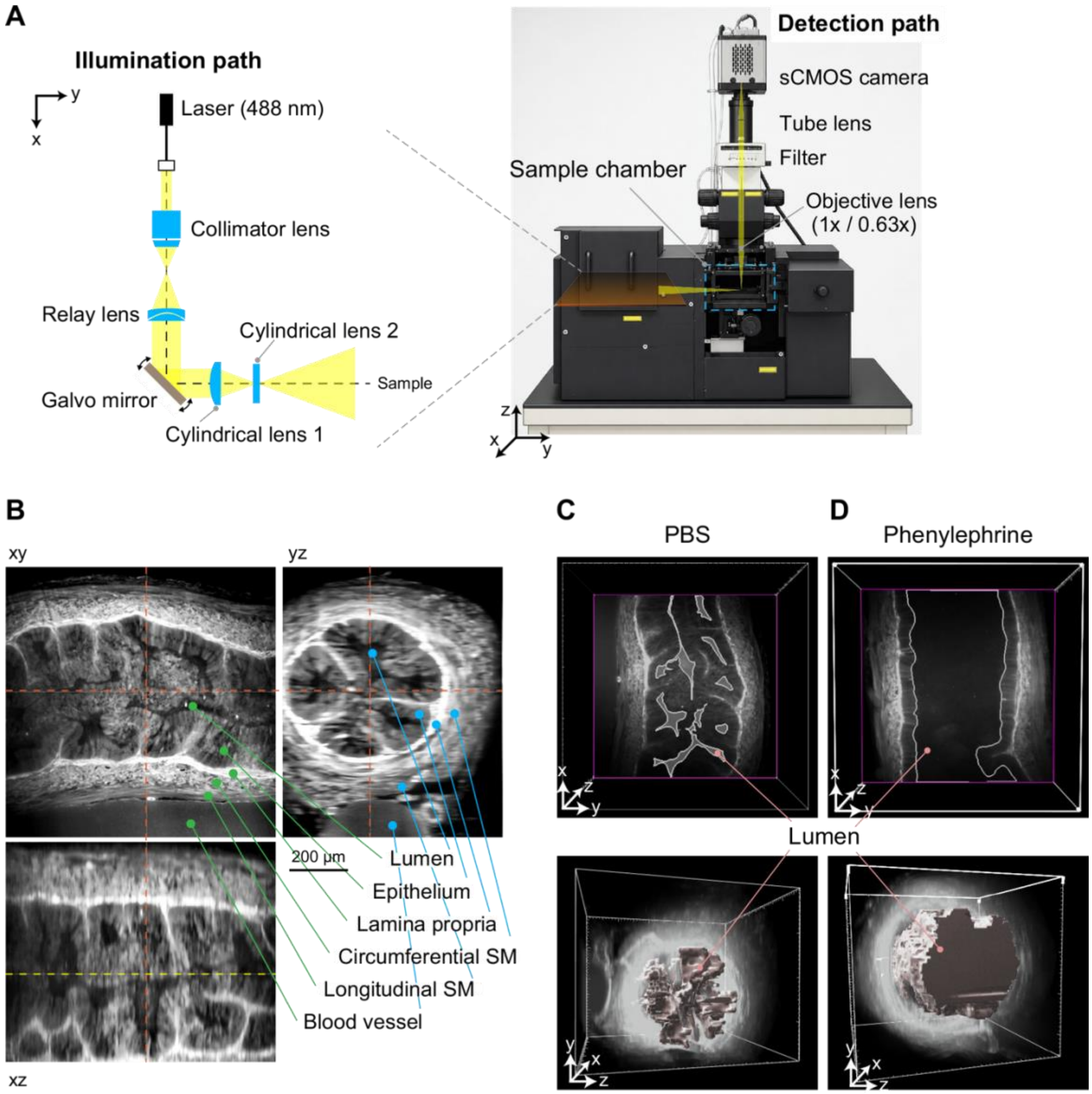

Fig. S2. Light-sheet microscopy and whole-tissue imaging

(A) Schematic overview of the custom MVX10-based light-sheet fluorescence microscopy system. Overall configuration of the custom-built light-sheet fluorescence microscopy system constructed around an MVX10 macro zoom microscope platform. The left panel illustrates the illumination path, and the right panel shows the detection path and sample chamber. In the illumination path, the 488 nm laser beam is first passed through a collimator lens to generate a collimated excitation beam. The beam is then directed through a relay lens and reflected by a galvo mirror, which rapidly sweeps the beam across the specimen to generate the scanning motion required for light-sheet formation. After reflection by the galvo mirror, the beam passes through two cylindrical lenses, each serving a distinct role in shaping the excitation profile. Cylindrical lens 1 focuses the excitation beam in one axis, producing a line focus, whereas cylindrical lens 2 further transforms the scanned beam into a laterally extended illumination profile at the sample plane. Together with the galvo scanning motion, these cylindrical optics generate a scanned light sheet at the specimen, following the principle of digitally scanned light-sheet fluorescence microscopy. In the detection path, fluorescence emitted from the illuminated plane is collected orthogonally through the objective lens and transmitted through the filter and tube lens to the camera. This orthogonal arrangement

spatially separates excitation and detection, enabling optical sectioning of the specimen with reduced out-of-focus background. The sample is positioned in a chamber at the intersection of the illumination and detection axes, where the scanned excitation beam forms the imaging plane. Arrows indicate the coordinate axes used to denote the optical geometry of the system. For the details, see the section Optical tissue clearing and light-sheet microscopy in Materials and Methods.

(B) Orthogonal view of the distal vas deferens in a Lifeact-EGFP mouse with annotations. Distinct tissue layers can be identified based on fluorescence intensity and texture: the lumen appears as a low-intensity central gap, the lamina propria as a thick layer with strong fluorescence, and the epithelial layer is located in between. Scale bar, 200 µm. The horizontal scale applies equally to the vertical dimension.

(C,D) Snapshots of Imaris-based 3D renderings of the distal wrinkle segment after PBS control treatment (C) and PE treatment (D). Corresponds to Movies 9 and 10, respectively.

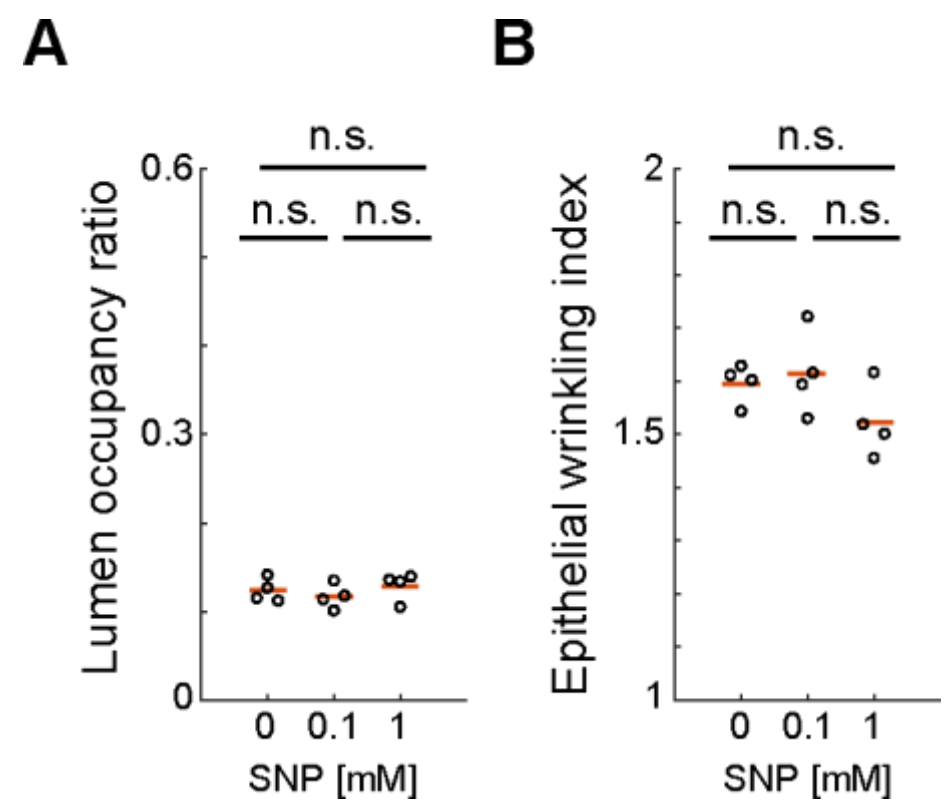

Fig. S3. Quantification of morphological changes in the isolated distal wrinkle segment under ex vivo conditions.

(A) Lumen occupancy ratio. N = 4; Kruskal-Wallis test, $p = 0.551$.

(B) Epithelial wrinkling index. N = 4; Kruskal-Wallis test, $p = 0.236$.

Movie S1 (separate file). In vivo PE-induced vas deferens contraction and sperm transport in a wild-type mouse, corresponding to Figure 1B.

Movie S2 (separate file). In vivo PE-induced vas deferens contraction and sperm transport in a *Pih1d3* knockout mouse, related to Figure 1B.

Movie S3 (separate file). Ex vivo PE-induced vas deferens contraction and sperm transport in a wild-type mouse, corresponding to Figure 1D.

Movie S4 (separate file). Ex vivo PE-induced vas deferens contraction and sperm transport in a *Pih1d3* knockout mouse, related to Figure 1F.

Movie S5 (separate file). Intravital TPEM imaging of luminal sperm dynamics before PE treatment, corresponding to Figure 2B.

Movie S6 (separate file). Intravital TPEM imaging of luminal sperm dynamics two min after PE treatment, corresponding to Figure 2C.

Movie S7 (separate file). Intravital TPEM imaging of luminal sperm dynamics five min after PE treatment, corresponding to Figure 2D.

Movie S8 (separate file). Stereomicroscopy imaging of luminal sperm flow in the vas deferens during PE treatment, corresponding to Figure 2E.

Movie S9 (separate file). 3D rendering of the distal wrinkle segment after PBS control treatment, corresponds to Figure 3E.

Movie S10 (separate file). 3D rendering of the distal wrinkle segment after PE stimulation, corresponds to Figure 3F.

Movie S11 (separate file). Intravital TPEM imaging of lumen opening in the distal wrinkle region of the vas deferens, corresponding to Figure 4A.

Movie S12 (separate file). Intravital TPEM imaging of ROCK activity following PE treatment, corresponding to Figure 5A.

Movie S13 (separate file). Intravital TPEM imaging of PKA activity following PE treatment, corresponding to Figure 5B.

Movie S14 (separate file). Intravital TPEM imaging of ERK activity following PE treatment, corresponding to Figure 5C.
